# Phenotypic Displays of Cholinergic Enzymes Associate With Markers of Inflammation, Neurofibrillary Tangles, and Neurodegeneration in Pre- and Early Symptomatic Dementia Subjects

**DOI:** 10.3389/fnagi.2022.876019

**Published:** 2022-05-26

**Authors:** Unnur D. Teitsdottir, Taher Darreh-Shori, Sigrun H. Lund, Maria K. Jonsdottir, Jon Snaedal, Petur H. Petersen

**Affiliations:** ^1^Faculty of Medicine, Department of Anatomy, Biomedical Center, University of Iceland, Reykjavik, Iceland; ^2^Division of Clinical Geriatrics, Department of Neurobiology, Care Sciences and Society, Center for Alzheimer Research, Karolinska Institutet, Campus Flemingsberg, Stockholm, Sweden; ^3^deCODE genetics/Amgen, Inc., Reykjavik, Iceland; ^4^Department of Psychology, Reykjavik University, Reykjavik, Iceland; ^5^Department of Psychiatry, Landspitali-National University Hospital, Reykjavik, Iceland; ^6^Memory Clinic, Department of Geriatric Medicine, Landspitali-National University Hospital, Reykjavik, Iceland

**Keywords:** Alzheimer’s disease, cerebrospinal fluid, acetylcholinesterase, butyrylcholinesterase, cholinergic system, inflammation, neurodegenaration, biomarkers

## Abstract

**Background:**

Cholinergic drugs are the most commonly used drugs for the treatment of Alzheimer’s disease (AD). Therefore, a better understanding of the cholinergic system and its relation to both AD-related biomarkers and cognitive functions is of high importance.

**Objectives:**

To evaluate the relationships of cerebrospinal fluid (CSF) cholinergic enzymes with markers of amyloidosis, neurodegeneration, neurofibrillary tangles, inflammation and performance on verbal episodic memory in a memory clinic cohort.

**Methods:**

In this cross-sectional study, 46 cholinergic drug-free subjects (median age = 71, 54% female, median MMSE = 28) were recruited from an Icelandic memory clinic cohort targeting early stages of cognitive impairment. Enzyme activity of acetylcholinesterase (AChE) and butyrylcholinesterase (BuChE) was measured in CSF as well as levels of amyloid-β_1–42_ (Aβ_42_), phosphorylated tau (P-tau), total-tau (T-tau), neurofilament light (NFL), YKL-40, S100 calcium-binding protein B (S100B), and glial fibrillary acidic protein (GFAP). Verbal episodic memory was assessed with the Rey Auditory Verbal Learning (RAVLT) and Story tests.

**Results:**

No significant relationships were found between CSF Aβ_42_ levels and AChE or BuChE activity (*p* > 0.05). In contrast, T-tau (*r* = 0.46, *p* = 0.001) and P-tau (*r* = 0.45, *p* = 0.002) levels correlated significantly with AChE activity. Although neurodegeneration markers T-tau and NFL did correlate with each other (*r* = 0.59, *p* < 0.001), NFL did not correlate with AChE (*r* = 0.25, *p* = 0.09) or BuChE (*r* = 0.27, *p* = 0.06). Inflammation markers S100B and YKL-40 both correlated significantly with AChE (S100B: *r* = 0.43, *p* = 0.003; YKL-40: *r* = 0.32, *p* = 0.03) and BuChE (S100B: *r* = 0.47, *p* < 0.001; YKL-40: *r* = 0.38, *p* = 0.009) activity. A weak correlation was detected between AChE activity and the composite score reflecting verbal episodic memory (*r* = −0.34, *p* = 0.02). LASSO regression analyses with a stability approach were performed for the selection of a set of measures best predicting cholinergic activity and verbal episodic memory score. S100B was the predictor with the highest model selection frequency for both AChE (68%) and BuChE (73%) activity. Age (91%) was the most reliable predictor for verbal episodic memory, with selection frequency of both cholinergic enzymes below 10%.

**Conclusions:**

Results indicate a relationship between higher activity of the ACh-degrading cholinergic enzymes with increased neurodegeneration, neurofibrillary tangles and inflammation in the stages of pre- and early symptomatic dementia, independent of CSF Aβ_42_ levels.

## Introduction

The neuropathological changes in AD include the accumulation of beta-amyloid (Aβ) in plaques and hyper-phosphorylated tau protein in neurofibrillary tangles (NFT), leading to loss of synapses, dendrites, and eventually neurons. The most consistent neuronal loss through the progression of AD is found within the cholinergic system of the basal forebrain (Mufson et al., 2003; Giacobini et al., 2022). The cholinergic neurons of this region are the major source of cholinergic innervation to the cerebral cortex and hippocampus, playing a pivotal role in cognitive functions including memory, learning and attention (Ballinger et al., 2016). Due to the critical role of the neurotransmitter acetylcholine (ACh) in cognitive functions, most of the approved pharmacological treatments for AD are cholinesterase inhibitors (ChEIs). ChEIs increase the availability of ACh at synapses in the brain and are among the few drugs that have been proven clinically useful in the treatment of AD, thus validating the cholinergic system as an important therapeutic target in the disease (Hampel et al., 2018).

According to the classical view of cholinergic signaling (Adem, 1992), the neurotransmitter ACh is synthesized in the cytoplasm of cholinergic neurons by the enzyme choline acetyltransferase (ChAT). ACh molecules are released into the synaptic clefts for initiating or propagating neurotransmission. The transmission is subsequently terminated by the cleavage of ACh by cholinesterases, acetylcholinesterase (AChE) and butyrylcholinesterase (BuChE). Notably, ACh is not restricted to neurons and synapses. The cholinergic signaling system has also been associated with inflammation, both generally and in neurodegenerative diseases like AD. ACh is hypothesized to act as a suppressor on non-excitable cholinoceptive cells, including astrocytes and microglia, inhibiting cytokine release through activation of nicotinic α7-ACh receptors (Van Westerloo et al., 2005; Pavlov et al., 2009; Benfante et al., 2021). Recent research demonstrated that astrocytes secrete the ACh synthesizing enzyme ChAT, suggesting that the physiological function of extracellular ChAT is to maintain a steady-state equilibrium of hydrolysis and synthesis of ACh (Vijayaraghavan et al., 2013).

Extracellular accumulation of Aβ in AD may cause imbalances in cholinergic signaling, which facilitates increased degradation of ACh and enhanced cytokine expression and release. Such an exaggerated inflammatory microenvironment could, in turn, have consequential neurodegenerative effects (Malmsten et al., 2014). BuChE may also have a particularly critical role in the dynamic control of levels of extracellular ACh, *via* its ACh hydrolyzing activity, as the primary source of it in the CNS is attributed to non-excitable cells such as astrocytes and microglia (Mesulam et al., 2002; Revathikumar et al., 2016). In plasma, increased AChE activity has been associated with AD, although results have been inconsistent (García-Ayllón et al., 2010).

In recent years, a paradigm shift has occurred from clinical to biological definition of AD based on *in vivo* biomarkers measured in cerebrospinal fluid (CSF) or with positron emission tomography (PET) imaging. In 2018, the National Institute on Aging and Alzheimer’s Association (NIA-AA) (Jack et al., 2018) created a research framework for AD diagnosis, defining AD based on biomarker evidence of pathology. Understanding how biomarkers reflecting different biological processes could influence AD pathogenesis and severity is critical for the improvement of diagnosis and development of effective pharmacologic treatments. It is essential for the evaluation of novel biomarkers to examine their relationship with signature AD pathology, independent of diagnosis. Such an approach could both enhance understanding of the underlying pathology of AD and the sequence of events leading to cognitive impairment. The aim of this study was to examine the association between the activity of cholinergic enzymes and CSF markers reflecting the state of brain amyloidosis (Aβ_42_), neurodegeneration (T-tau, NFL), neurofibrillary tangles (P-tau) and inflammation (YKL-40, S100B, GFAP) among subjects at the pre-and early symptomatic stages of dementia. The second aim was to explore the relationships of the same enzymes with the loss of verbal episodic memory.

## Materials and Methods

### Subjects

**Figure 1:**
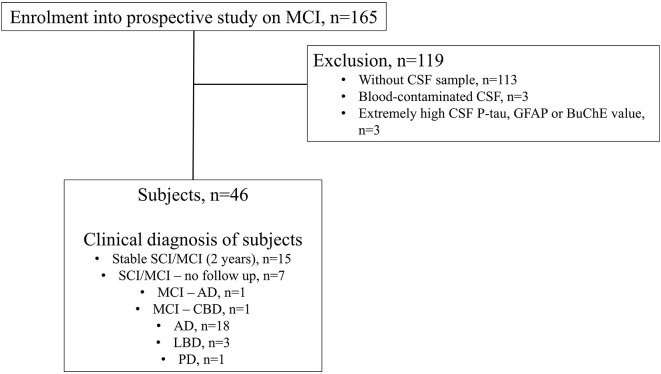
Flow chart illustrating the inclusion/exclusion criteria for the study and clinical diagnosis of subjects.

The current study cohort and the selection criteria have been described earlier (Teitsdottir et al., 2020, 2021). Subjects were recruited from the Icelandic MCI study cohort (*n* = 165). The cohort was comprised of individuals who had been referred to Landspitali University Hospital (LUH) Memory Clinic over a four-year period. The inclusion criteria for joining the cohort study were: (i) a score between 24 and 30 on the Mini-Mental State Examination (MMSE) (Folstein et al., 1975); and (ii) a score of 4.0 or less on the Informant Questionnaire on Cognitive Decline in the Elderly (IQCODE) (Jorm, 1994). The exclusion criteria were the following: (i) cognitive impairment caused by a pre-existing condition; (ii) difficulties participating due to health or social issues; and (iii) residency outside the Reykjavík Capital Area. Each subject underwent various measurements at baseline, which included a medical assessment and a detailed neuropsychological assessment as well as brain magnetic resonance imaging (MRI) for the evaluation of medial temporal lobe atrophy (MTA), global cortical atrophy (GCA) and white matter lesions (Fazekas). Lumbar puncture was carried out for the collection of CSF, but the intervention was optional by the requirement of the National Bioethics Committee. For this cross-sectional study, the final sample included 46 subjects (Figure 1). Only those who underwent lumbar puncture were selected from the Icelandic MCI study cohort, excluding 113 subjects. In addition, six other subjects were removed due to excessively high CSF P-tau (*n* = 1), GFAP (*n* = 1), AChE and BuChE (*n* = 1) values or blood-contamination in CSF samples (*n* = 3). Clinical diagnosis of AD was based on the criteria for probable AD dementia defined by the National Institute on Aging-Alzheimer’s Association (NIA-AA) (Mckhann et al., 2011), with evidence of AD pathophysiological processes (based on MTA score or/and analysis of core CSF markers). Patients with Lewy body dementia (LBD) were diagnosed based on the consensus criteria of McKeith (Mckeith et al., 2017). The diagnosis of MCI required the fulfillment of the Winblad criteria (Winblad et al., 2004). Those without cognitive impairment were considered to have subjective mild cognitive impairment (SCI), as they had been referred to the Memory Clinic due to concerns of cognitive decline. Of the 46 subjects in this study, 15 were diagnosed with SCI or MCI and remained stable after a two-year follow-up. One subject converted from a diagnosis of MCI to AD after a two-year follow-up and another one from MCI to cortico-basilar degeneration (CBD) after a one-year follow-up. A total of 18 subjects were diagnosed with AD, three with LBD and one with Parkinson’s disease (PD) at baseline. Seven of the subjects were diagnosed with SCI and MCI at baseline but left the study before one year or two-year follow-up. None of the subjects had been prescribed cholinergic drugs before entering or were prescribed them during the study.

### CSF Collection and Analysis

The collection of CSF and the measurements of protein concentrations have previously been described (Teitsdottir et al., 2020, 2021). Collection of CSF was done via lumbar puncture with a 22-gauge spinal needle at the L3/4 or L4/5 interspace. Samples, uncentrifuged, were frozen in 2 ml polypropylene tubes and stored at −80°C. Levels of all proteins were determined using commercially available sandwich enzyme-linked immunosorbent assays (ELISAs) and performed according to manufacturer’s instructions. Levels of T-tau (IBL International, Hamburg, Germany), P-tau181 (INNOTEST, Gent, Belgium), and Aβ_42_ (IBL International, Hamburg, Germany), were measured in the ISO 15189 accredited medical laboratory MVZ Labor P.D. Dr. Volkmann und Kollegen GbR (Karlsruhe, Germany). Levels of NFL (Uman Diagnostics, Umeå, Sweden), YKL-40 (Quantikine ELISA Human Chitinase-3-like 1; R&D systems, MN, USA), S100B (BioVendor GmbH, Heidelberg, Germany) and GFAP (BioVendor GmbH, Heidelberg, Germany) were measured in a laboratory at the University of Iceland. All assays had mean Intra-assay CV <10% and Inter-assay CV <15%. The activity of BuChE and AChE in CSF was measured as described in Darreh-Shori et al. (2006, 2008).

### Subject Grouping Based on CSF Measures

Three different CSF biomarker profiles were created, each based on the levels of one core CSF biomarker (Aβ_42_ T-tau and P-tau). The CSF profile was divided into two categories, abnormal (positive) or normal (negative). The specific cut-off points were established by MVZ Labor P.D. Dr. Volkmann und Kollegen GbR, the laboratory performing the ELISAs. Abnormal values were defined as Aβ_42_ < 375 pg/ml, T-tau > 445 pg/ml and P-tau > 61 pg/ml.

### Neuropsychological Tests

A detailed neuropsychological assessment was carried out by licensed psychologists under the guidance of a clinical neuropsychologist. Two tests were used for the evaluation of verbal episodic memory, The Rey Auditory Verbal Learning Test (RAVLT) (Lezak et al., 2012), and a Story test based on the Logical Memory test of the Wechsler Memory Scale-Revised (Wechsler, 1987). RAVLT requires the subject to learn 15 nouns presented across five consecutive trials. Each trial is followed by a free-recall test (immediate recall). The sum of the number of words recalled from trials 1 through 5 (0–75 points) is calculated for the score of RAVLT immediate recall. After a 30 min delay, the subject is asked to recall as many words as possible without hearing them again (delayed recall). The score is a sum of correctly recalled words (0–15 points). Lastly, a list containing the previous 15 nouns as well as 30 new ones, is read to the subject whose task is to recognize the nouns from the list, with number of false positives subtracted from the score (−30–15 points). The second test is composed of a brief story, presented orally by the examiner. The story includes 25 story ideas, where the subject is required to repeat it immediately after presentation without any clues given. A point is given for the number of story ideas correctly recalled (0–25 points). After a 30-min delay, the subject is asked to recall the story again without it being orally repeated again.

### Statistical Analysis

Descriptive group comparisons were performed using Mann-Whitney U tests or Kruskal-Wallis H tests for continuous variables and chi-square tests for categorical variables. The composite score for verbal episodic memory was calculated by averaging the *z*-scores of each neuropsychological test and subsequently converting those scores into *z*-scores. Raw values of CSF proteins (Aβ_42_, P-tau, T-tau, NFL, YKL-40, S100B, GFAP) were naturally log-transformed to account for a non-normal distribution. Pearson’s correlations and scatter plots were used for estimation and visualization of relationships between continuous variables. Least absolute shrinkage and selection operator (LASSO) regression combined with stability selection was applied for the identification of stable predictors in multivariable models (Tibshirani, 1996). The function stabsel from the package stabs in R was implemented for the performance of stability selection, which utilized the package glmnet for LASSO model fitting on each subsample (Meinshausen and Bühlmann, 2010; Hofner et al., 2015). A total of 100 subsamples were drawn, with each being half the size of the original sample. The cut-off value of 75% was chosen as a criterion for stable selection (calculated as the percentage frequency of a variable being selected into a model) and per-family error rate (PFER) was set to 1. All statistical analyses were performed using R (version 3.6.1, The R Foundation for Statistical Computing).

## Results

### Study Cohort Characteristics

Table 1 shows the demographic, pathophysiological and clinical characteristics of the cohort by CSF Aβ_42_ and T-tau profiles. No statistical difference (*p* > 0.05) was found in characteristics between subjects with abnormally low CSF Aβ_42_ levels (positive profile; +) and those with normal Aβ_42_ levels (negative profile; -). In contrast, statistical difference was found between the CSF T-tau profile groups. Subjects with abnormally high CSF T-tau levels (+) were statistically higher in age (*p* = 0.02), CSF NFL levels (*p* < 0.001) and CSF YKL-40 levels (*p* = 0.02) compared to those with normal T-tau levels (-). The T-tau+ group did also score significantly lower on all neuropsychological subtests reflecting verbal episodic memory (*p* < 0.01).

**Table 1:** Subject demographics, CSF marker levels, and cognitive scores by CSF Aβ_42_ and T-tau profiles.

	**CSF Aβ_42_ profile**			**CSF T-tau profile**		
	**Aβ_42_−**	**Aβ_42_+,**	***p* value**	**T-tau−,**	**T-tau+,**	***p* value**
	**Aβ_42_ >**	**Aβ_42_ <**	**a**	**T-tau <**	**T-tau >**	**a**
	**375 pg/ml**	**375 pg/ml**		**445 pg/ml**	**445 pg/ml**	
	**(*n* = 33)**	**(*n* = 13)**		**(*n* = 29)**	**(*n* = 17)**	
**Demographics**						
Gender (M/F)	13/20	8/5	0.18	14/15	7/10	0.64
Age, years	71 (46–85)	70 (51–84)	0.75	70 (46–85)	77 (51–84)	0.02
Education, years	13 (6–19)	13 (6–20)	0.77	13 (6–19)	13 (6–20)	0.75
**Clinical diagnosis**						
Stable SCI or MCI/AD^b^/						
OD^c^/SCI or MCI—no follow-up	12/11/5/5	3/8/0/2	N/A^d^	15/4/3/7	0/15/2/0	N/A^d^
**CSF measures**						
Aβ_42_ (pg/ml)	671 (404–1434)	254 (140–335)	N/A	594 (167–1434)	490 (140–977)	0.27
T-tau (pg/ml)	294 (106–1886)	397 (132–916)	0.09	253 (106–438)	643 (475–1086)	N/A
P-tau (pg/ml)	57 (33–144)	83 (30–125)	0.40	49 (30–87)	106 (70–144)	N/A
AChE activity (nmol/min/ml)	12.2 (4.47–16.3)	10.5 (8.0–16.0)	0.35	11.1 (4.4–15.5)	13.5 (8.0–16.3)	0.008
BuChE activity (nmol/min/ml)	6.3 (4.2–10.9)	6.4 (4.7–11.5)	0.92	6.2 (4.2–9.4)	7.0 (4.7–11.5)	0.11
NFL (ng/ml)	2.1 (1.0–5.0)	2.3 (1.2–5.3)	0.60	2.0 (1.0–3.6)	3.0 (1.6–5.3)	<0.001
YKL-40 (ng/ml)	194 (83–367)	177 (124–351)	0.87	160 (83–365)	235 (124–367)	0.02
S100B (pg/ml)	233 (143–458)	240 (175–509)	0.89	228 (143–382)	243 (175–509)	0.15
GFAP (ng/ml)	1.2 (0.2–21.3)	1.4 (0.8–5.8)	0.14	1.3 (0.2–6.3)	1.1 (0.5–21.3)	0.59
**Global cognition**						
MMSE, score	28 (24–30)	27 (24–30)	0.32	28 (24–30)	27 (24–30)	0.25
**Verbal episodic memory**						
RAVLT immediate recall, score^e^	30 (13–65)	26 (15–51)	0.27	31 (16–65)	25 (13–39)	0.009
RAVLT delayed recall, score^e^	3 (0–15)	2 (0–12)	0.13	4 (0–15)	1 (0–8)	0.003
RAVLT recognition-fp, score^f^	7 (−3–15)	6 (0–15)	0.61	7 (2–15)	4 (−3–9)	0.001
Story immediate recall, score	9 (1–21)	6 (2–18)	0.21	11 (2–21)	6 (1–14)	0.003
Story delayed recall, score	7 (0–19)	6 (0–16)	0.30	8 (0–19)	3 (0–12)	0.002
Composite *z*-score^f^	−0.1 (−1.5–2.3)	−0.5 (−1.2–1.9)	0.13	0.1 (−1.0–2.3)	−0.7 (−1.5–−0.2)	<0.001

*AD, Alzheimer’s disease; CSF, Cerebrospinal fluid; MCI, Mild Cognitive Impairment; MMSE, Mini-Mental State—Examination; N/A, Not applicable; OD, Other dementia; RAVLT, Rey Auditory-Verbal Learning Test; SCI, Subjective Cognitive Impairment. Values are shown as median (range) or as numbers per group, ^a^Mann-Whitney U non-parametric tests used for continuous variables and Chi-Square tests for categorical variables. ^b^The AD category included one subject who converted from MCI to AD. ^c^The OD category included one subject who converted from MCI to OD. *P*-values not applicable for ^d^clinical diagnosis due to CSF Aβ_42_ and Tau values being a part of the diagnostic criteria for AD. Analysis based on 45^e^ or 44^f^ subjects*.

**Figure 2:**
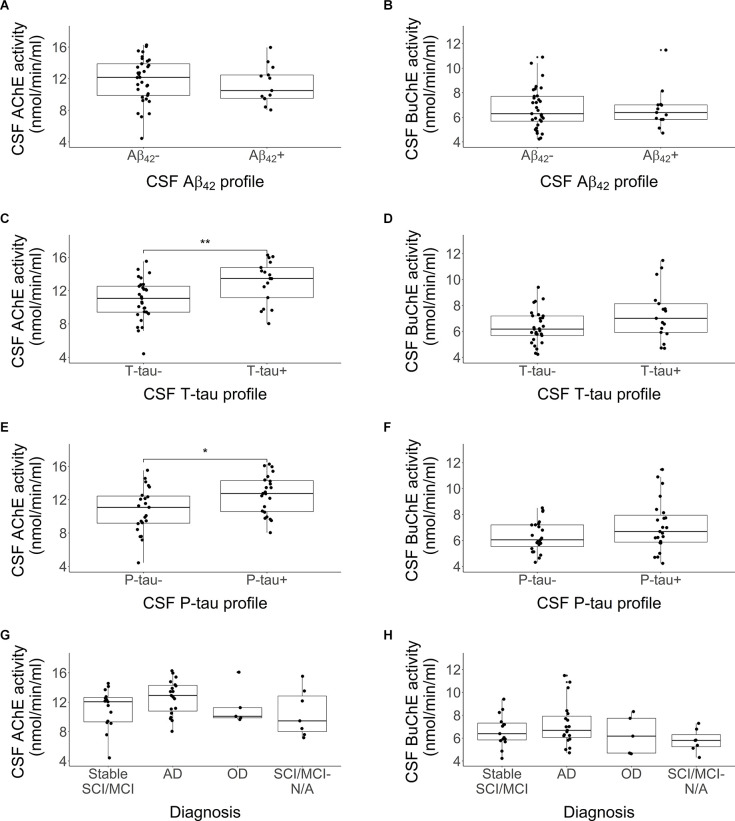
Comparison in activity of CSF AChE and BuChE enzymes by CSF Aβ_42_ profile **(A,B)**, T-tau profile **(C,D)**, P-tau profile **(E,F)**, and clinical diagnosis **(G,H)**, *p < 0.05, **p < 0.01. Each profile was divided into two categories, abnormal (+) and normal (-) values. Abnormal values were defined as Aβ_42_ < 375 pg/ml, T-tau > 445 pg/ml and P-tau > 61 pg/ml. The lower and upper horizontal lines of the boxplot correspond to the 25th centile and 75th centile and the middle line to the median. CSF AChE activity was significantly higher among subjects with a CSF T-tau+ or P-tau+ profiles compared to those without.

Distributions in cholinergic activity (AChE and BuChE) between CSF profiles (Aβ_42_, T-tau and P-tau) and clinical diagnosis are visualized with boxplots in Figure 2. No significant differences were detected in AChE (Figure 2A; *p* = 0.35) or BuChE activity (Figure 2B; *p* = 0.92) between the Aβ+ and Aβ- profile groups. In contrast, AChE activity was significantly higher among subjects with T-tau+ profile compared to those with a T-tau- profile (Figure 2C; *p* = 0.008). No differences were detected between BuChE activity between the same groups (Figure 2D; *p* = 0.11). The same pattern was observed as well for P-tau, with higher AChE activity found among the positive group compared to the negative one (Figure 2E; *p* = 0.02), and no difference between groups in regard to BuChE activity (Figure 2F; *p* > 0.05). No significant difference was observed in AChE (Figure 2G; *p* > 0.05) or BuChE activity (Figure 2H; *p* > 0.05) between different diagnostic groups.

### Correlation Matrix Between CSF Markers, Age, and Education

**Figure 3:**
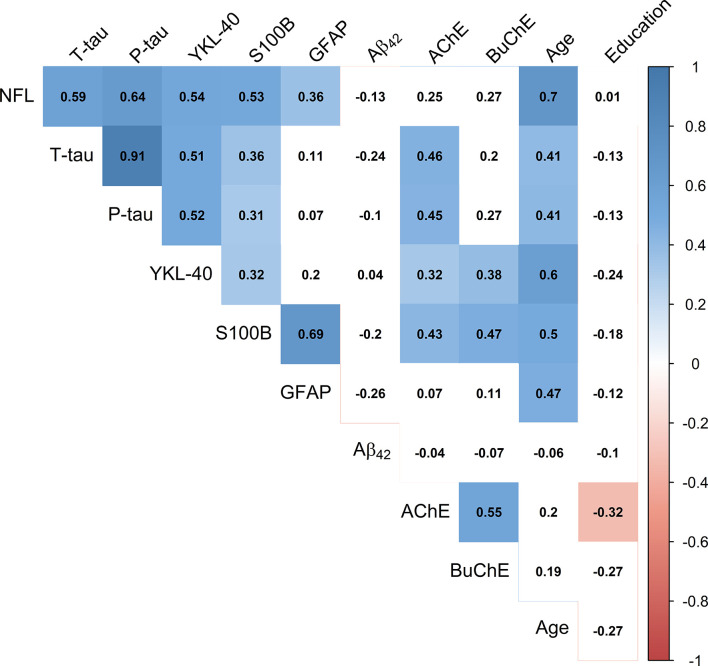
Correlation matrix depicting Pearson’s coefficients between CSF activity of cholinergic enzymes, CSF protein levels, age and education. Colored squares indicate statistical significance (*p* < 0.05). Blue and red colors present positive and negative coefficients, respectively. Values of CSF proteins (Aβ_42_, T-tau, P-tau, NFL, YKL-40, S100B, and GFAP) were natural log-transformed.

Pearson’s correlations between the CSF markers, age, and education are presented in Figure 3. Inflammatory markers YKL-40 and S100B and neurodegeneration markers NFL, P-tau and T-tau all correlated positively and significantly (*p* < 0.05) with each other. GFAP did only significantly correlate with the CSF markers S100B (*r* = 0.69, *p* < 0.001) and NFL (*r* = 0.36, *p* = 0.01). No CSF marker correlated significantly with Aβ_42_ (*p* > 0.05). All the CSF markers, except for Aβ_42_ and the cholinergic enzymes, correlated positively with age (*p* < 0.05). AChE activity correlated significantly with levels of P-tau, T-tau, YKL-40, and S100B (*p* < 0.05), but not with levels of NFL (*r* = 0.25, *p* = 0.09). BuChE activity correlated significantly with levels of YKL-40 and S100B (*p* < 0.01), but not with P-tau (*r* = 0.27, *p* = 0.07), T-tau (*r* = 0.20, *p* = 0.18) or NFL (*r* = 0.27, *p* = 0.06).

**Figure 4:**
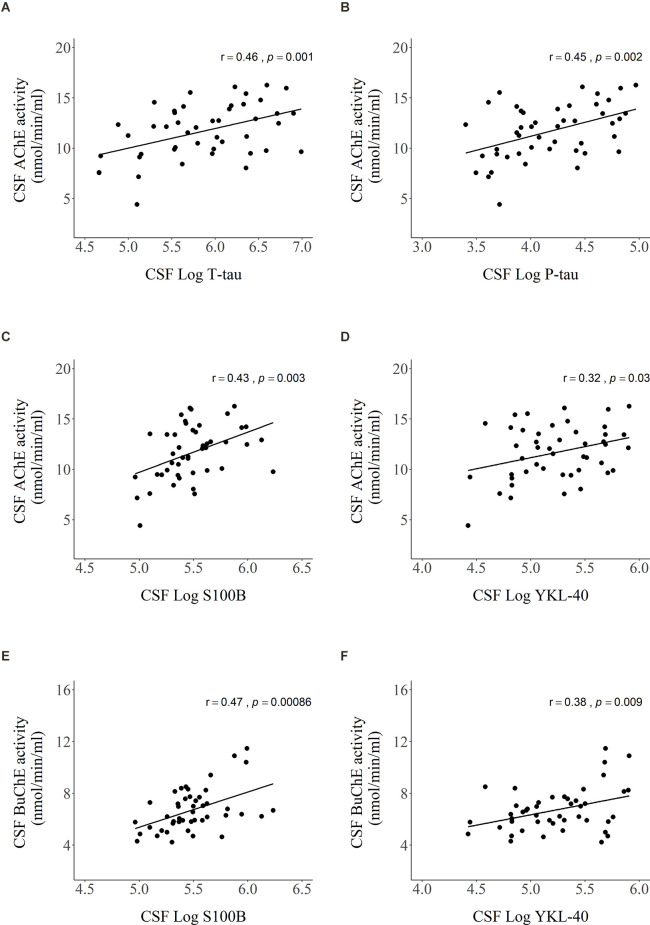
Scatter plots presenting Pearson’s correlations between AChE activity and levels of **(A)** T-tau, **(B)** P-tau, **(C)** S100B, **(D)** YKL-40, and BuChE activity and levels of **(E)** S100B and **(F)** YKL-40 in CSF. Values of CSF proteins (T-tau, P-tau, YKL-40, and S100B) were natural log-transformed.

Significant relationships of CSF AChE and BuChE activities with CSF markers from Figure 3 are visualized by scatter plots in Figure 4. Moderately strong, positive correlations were detected between AChE activity and levels of: (A) T-tau (*r* = 0.46, *p* = 0.001), (B) P- tau (*r* = 0.45, *p* = 0.002), and (C) S100B (*r* = 0.43, *p* = 0.003). Weak correlation was found between AChE activity and (D) YKL-40 levels (*r* = 0.32, *p* = 0.03). As with AChE, BuChE also showed moderately strong, positive correlation with levels of (E) S100B (*r* = 0.47, *p* < 0.001) and (F) YKL-40 (*r* = 0.38, *p* = 0.009).

### Selection of Best Predictors for Cholinergic Enzyme Activity

**Figure 5:**
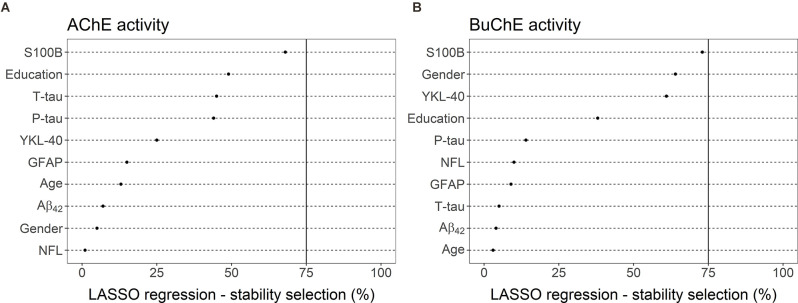
LASSO linear regression—stability selection analyses for the identification of a set of variables predicting CSF enzyme activities of **(A)** AChE and **(B)** BuChE with the highest consistency. The cut-off selection value was set at 75% and the per-family error rate (PFER) at 1. Variables selected into the model at least 75% of the time were considered reliable predictors. None of the variables reached that criterion, neither for the prediction of AChE nor BuChE activity.

LASSO linear regression with a stability selection was performed for the identification of a set of variables predicting cholinergic enzyme activity with the highest consistency (Figure 5). Variables with stability selection above 75% were considered reliable predictors. Two analyses were performed, one for each enzyme (AChE and BuChE) as a dependent variable. Ten possible predictors could be selected for each analysis, five CSF markers (Aβ_42_, T-tau, P-tau, NFL, YKL-40, S100B, GFAP) and three demographic measures (gender, age, and length of education).

No variables reached the selection criteria for prediction of AChE activity (Figure 5A). S100B was the predictor with the highest selection frequency (68%), by far the highest compared to other measures. The next predictors in order of selection frequency were education (49%), T-tau (45%), and P-tau (44%). All other possible predictors had much lower selection frequency (≤25%).

S100B was also the measure most often selected into a model (73%) for the best prediction of BuChE activity (Figure 5B). The CSF marker almost reached the selection criteria of 75%. Gender (64%) and YKL-40 (61%) both had selection frequency over 60%. Education had the fourth highest frequency (38%). All other possible predictors had far lower selection frequency (≤15%).

### Association Between CSF Markers and Verbal Episodic Memory

**Figure 6:**
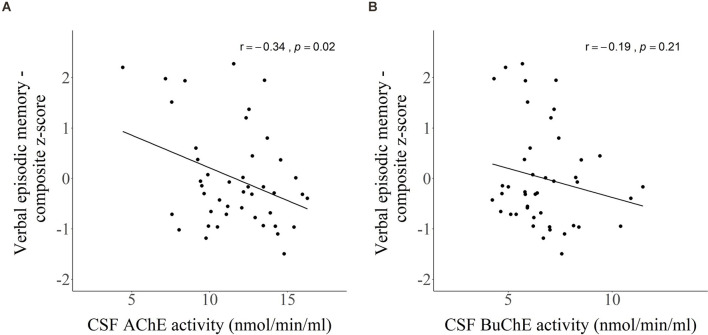
Scatter plots presenting Pearson’s correlations between CSF activity of **(A)** AChE and **(B)** BuChE with composite *z*-score of verbal episodic memory.

CSF activity of AChE (Figure 6A) showed a weak, negative correlation with the composite z-score reflecting verbal episodic memory (*r* = −0.34, *p* = 0.02), while BuChE (Figure 6B) did not reach significance (*r* = −0.19, *p* = 0.21). The analysis was based on 44 subjects as two subjects did not take the RAVLT test.

**Figure 7:**
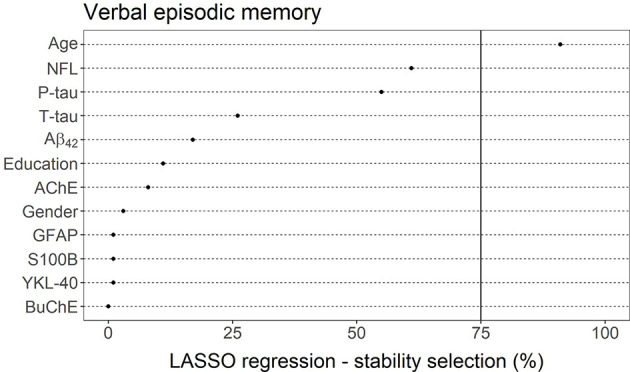
LASSO linear regression—stability selection analysis for the identification of a set of variables predicting verbal episodic memory performance. The cut-off selection value was set at 75% and the per-family error rate (PFER) at 1. Variables selected into the model at least 75% of the time were considered reliable predictors. Only age reached that criterion.

LASSO linear regression with a stability selection was also applied for identifying a set of variables (CSF markers and demographic variables) predicting verbal episodic memory composite score (Figure 7). Only age was selected as a reliable predictor, with a selection frequency of 91%. CSF markers NFL (61%) and P-tau (55%) both had a selection frequency above 50%. All other measurements had selection frequencies below 30%, including AChE (8%) and BuChE (0%).

## Discussion

We explored the association of cholinergic enzyme activity with CSF markers reflecting amyloidosis, neurodegeneration, neurofibrillary tangles and inflammation as well as with loss of verbal episodic memory among subjects in a memory clinic cohort. Our results indicated no difference in AChE and BuChE activity between individuals with abnormal and normal Aβ_42_ levels. In contrast, individuals with abnormally high T-tau or P-tau levels had increased AChE activity compared to those with normal levels. Higher levels of inflammatory markers S100B and YKL-40 associated with higher activity of AChE and BuChE. Interestingly, S100B was also the most reliable predictor of both AChE and BuChE activity in comparison to other CSF markers and demographic measures. Weak association was detected between AChE activity and verbal episodic memory performance, although the enzyme did not prove to be a reliable predictor when other measures had also been accounted for. Overall, these results indicate a relationship between higher activity of the ACh-degrading cholinergic enzymes and increased inflammation, accumulation of neurofibrillary tangles and neurodegeneration in the stages of pre- and early symptomatic dementia, independent of CSF Aβ_42_ levels.

The relationship between Aβ protein and cholinergic dysfunction has long been established through *in vitro* and post-mortem studies. AChE activity is consistently increased in regions around Aβ plaques and NFTs at all stages of the disease, although overall AChE activity decreases in the AD brain (Perry et al., 1980; Mesulam and Asuncion Morán, 1987; Ulrich et al., 1990). Studies have also reported about 40%–50% reduction in ACh synthesis in cultured cholinergic neurons upon exposure to a high nanomolar concentration of Aβ_42_ (Pedersen et al., 1996; Hoshi et al., 1997; Kar et al., 1998; Nunes-Tavares et al., 2012). More recent research indicate that Aβ peptides act directly as allosteric modulators of cholinergic signaling by forming highly stable and soluble complexes with apolipoprotein E (ApoE) and cholinesterases (Darreh-Shori et al., 2011a, b; Vijayaraghavan et al., 2013; Kumar et al., 2016). A better understanding of the functional relationship between Aβ and the cholinergic system is though needed, as the biochemical environment *in vivo* is complex. Results regarding association between Aβ_42_ levels and AChE activity in CSF have, for example, been inconsistent (Darreh-Shori et al., 2006; García-Ayllón et al., 2008; Johansson et al., 2013). Darreh-Shori et al. (2006) did not detect a relationship between Aβ_42_ levels and AChE activity among patients with mild AD. Similarly, Johansson et al. (2013) did not detect an association between same measures among AD patients. In contrast, García-Ayllón et al. (2008) did observe an association between Aβ_42_ levels and AChE activity within same patient group. Johansson et al. (2013) also did find a positive relationship between the proteins, but only within the whole study population (AD, other dementias, stable MCI and healthy controls). Neither Darreh-Shori et al. (2006) nor Gabriel et al. (2017) detected relationship between BuChE activity and Aβ_42_ levels among AD patients. Gabriel et al. (2017) did, however, detect a positive association among those patients carrying the ApoE-ε4 allele. In this current study, relationships between the activities of the cholinergic enzymes and Aβ_42_ levels were not detected.

Previous post-mortem studies have shown that the loss of cortical cholinergic innervation is associated with, and potentially caused by, NFT in the nucleus basalis of Meynert (NBM) of the basal forebrain (Geula and Mesulam, 1995; Braak and Del Tredici, 2013; Mesulam, 2013), with degeneration already present at the very early stages of AD (Mesulam et al., 2004; Mufson et al., 2008). Silveyra et al. (2012) reported that P-tau could be an important regulator of AChE expression. Over-expression of P-tau in transgenic mice (Tg-VLW) led to an increase in the activity of AChE, suggesting that increase in AChE expression around NFTs could be a consequence of disturbed tau phosphorylation. Very few studies have, however, examined the relations between CSF tau levels and cholinesterase activity in CSF. Our study found a correlation between T-tau and P-tau with AChE activity. This is in accordance with a study by Johansson et al. (2013), where a positive association between T-tau and P-tau levels with AChE activity was detected in CSF among cases of AD, other dementias, stable MCI and healthy controls. We did not find a correlation between T-tau or P-tau levels and BuChE activity. The results are in line with results from a study by Gabriel et al. (2017), which did not find correlations between CSF T-tau and P-tau with BuChE activity among AD patients. One possible explanation for the difference in results between the enzymes in regard to the neurodegeneration markers, could be due to localization. BuChE activity is mainly localized to glial cells while AChE is predominantly located within neurons and axons (Wright et al., 1993; Giacobini, 2003; Picciotto et al., 2012). Interestingly, we did not find a relationship between NFL, a marker of both neurodegeneration and white matter changes, and the activity of the cholinergic enzymes. NFL is considered a more general marker for neurodegeneration compared to T-tau, which has been associated with Aβ pathology (Mattsson et al., 2016). To the best of our knowledge, NLF has not been researched in regard to AChE and BuChE activity in CSF samples from a dementia cohort. A study by Aeinehband et al. (2015) among patients with multiple sclerosis (MS) found a positive correlation between NFL levels and BuChE activity, but not AChE activity.

The neurotransmitter ACh is not only restricted to neurons but can also act on regions distal from synaptic sites (Nizri et al., 2006). It has anti-inflammatory effects, inhibiting pro-inflammatory responses by acting on α7 nicotinic ACh receptors expressed on non-excitable cholinoceptive cells like astrocytes and microglia (Benfante et al., 2021). Extracellular ACh is therefore hypothesized to play a key role in homeostatic functions that include neuronal support, the maintenance of myelin, synaptic function and plasticity, Aβ clearance, and maintenance of the blood–brain barrier (Lane and He, 2013). Both AChE and BuChE enzymes play a major dynamic role in this homeostasis as they degrade ACh, with BuChE being specifically important for the function of glial cells as they serve as the enzyme’s primary source and location (Lane and He, 2013). S100B and GFAP are two commonly used CSF markers of astroglial reactivity. A study by Darreh-Shori et al. (2013) found that BuChE activity positively associated with S100B and GFAP in CSF among AD patients. A possible explanation could be that lower levels of extracellular ACh associate with higher BuChE activity, enhancing the reactivity of glia. This heightened function can be protective, but prolonged glial activation may gradually lead to degeneration and eventually loss of neurons, further triggering inflammatory responses (Lane and Darreh-Shori, 2015). No relationship was detected between AChE activity and levels of S100B and GFAP in the same study. Our study found a positive relationship between S100B, and to lesser extent YKL-40, with activity of both enzymes. To the best of our knowledge, YKL-40 has not been explored before in association with cholinergic enzymes. YKL-40 is widely used as a glial activation marker, although the cellular source of expression in brain remains uncertain. A variable pattern of YKL-40 expression that includes astrocytes, microglia or, on rare occasions, neurons have been associated with AD in studies (Querol-Vilaseca et al., 2017). Interestingly, we did not find association between GFAP levels with either enzyme. Different patterns of association between the enzymes with different inflammatory markers could possibly be explained by different cellular functions. Both YKL-40 (Bonneh-Barkay et al., 2012) and S100B (Donato et al., 2009) have extracellular functions while GFAP (Yang and Wang, 2015) is an intracellular protein. Increased GFAP levels in CSF may therefore be a later event in the pathogenic cascade.

Although all ChEIs enhance synaptic transmission, they exert their effects on cholinesterases differently. The drugs donepezil and galantamine are reversible inhibitors of AChE while rivastigmine is a pseudo-irreversible inhibitor of both AChE and BuChE. The difference in pharmacological properties affects both the ability of breaking down extrasynaptic ACh as well as the expression of extracellular AChE (Nordberg et al., 2009; Darreh-Shori and Soininen, 2010; Marucci et al., 2021). Therefore, different ChEIs could alter glial-neuronal interactions in different ways (Lane and Darreh-Shori, 2015). A larger, longitudinal study might be able to detect the effect of each ChEI on CSF markers reflecting cholinergic activity, inflammation, and neurodegeneration. Measures of those markers, in combination with factors including age, gender, and genotype, could potentially be of use in predicting which patients could benefit from a treatment with a specific ChEI.

It is unclear to what extent episodic memory dysfunction relates to structural and functional changes in the cholinergic system at the symptomatic pre-dementia stages (SCI or MCI; Peter et al., 2016). The basal forebrain undergoes severe neurofibrillary degeneration and neuronal loss in patients with moderate to severe AD, most prominent in the NBM. In comparison, changes at the MCI stage are thought to be characterized by alterations in cholinergic functions rather than by cholinergic neuronal loss (Schliebs and Arendt, 2011). In accordance with this, histological studies have revealed that cognitive deficits are not evident before at least 30% of the basal forebrain cholinergic neurons have degenerated (Schliebs and Arendt, 2006) and individuals with MCI show only around 15% volume loss compared to healthy controls (Peter et al., 2016). Our results are in line with those findings as only a weak association was found between AChE activity and verbal episodic memory performance. When other factors (e.g., age) were simultaneously considered, neither of the cholinergic enzymes proved to be a good predictor for verbal episodic memory. Previous studies have revealed NFL to be a promising progression marker, associating with cognitive impairment in both AD and LBD (Zetterberg et al., 2016; Olsson et al., 2019). In our study, the cholinergic enzymes did not correlate with levels of NFL, emphasizing further the lack of contribution of the cholinergic system to episodic memory dysfunction at the very early stages of dementia.

This study has several limitations. First, the results need to be validated in a larger study due to the sample being relatively small. Second, the association between variables could also possibly be underestimated as no healthy controls were included in the sample. Third, no genetic data was available for this study. It would have been of importance to explore the associations between different measures by genotype, especially BChE. The BChE-K variant has been linked to increased risk of developing AD, although results have been conflicting. In addition, a reduction of the BuChE activity in CSF of AD patients carrying both the K variant and the ApoE-ε4 allele has previously been reported (Darreh-Shori et al., 2012; Jasiecki and Wasąg, 2019; Jasiecki et al., 2019). However, the influence of the BChE-K variant on the levels of Aβ_42_, T-tau or P-tau in CSF is still unknown. Finally, a list of prescribed anticholinergic drugs was not available for the subjects of this study. This class of drugs binds to ACh receptors and blocks ACh neurotransmission. Anticholinergic burden refers to their cumulative effect and has been linked to adverse effects such as decline in cognitive function (Salahudeen et al., 2015; Lozano-Ortega et al., 2020). Therefore, the study could have benefited from including an anticholinergic burden score in the statistical analyses.

Our findings suggest that the activity of ACh-degrading cholinergic enzymes at the pre- and early symptomatic stages of dementia relate to inflammatory and neurodegenerative processes, but not to loss in verbal episodic memory as measured in this study. A better understanding of the cholinergic system and its relations to both pathology and cognitive functions are critical, given that it is the main target of current symptomatic treatment of AD. Further studies are needed for validation of these results, preferably with larger samples and access to genotype information.

## Data Availability Statement

The raw data supporting the conclusions of this article will be made available by the authors, without undue reservation.

## Ethics Statement

The studies involving human participants were reviewed and approved by the National Research Ethics Committee of Iceland (VSN-14-028). The study was conducted in accordance with the Helsinki Declaration latest revision of 2013. The patients/participants provided their written informed consent to participate in this study.

## Author Contributions

UT, TD-S, JS, and PP contributed to the conception and design of the study. UT and MJ contributed to the collection of data. UT wrote the first draft, performed the statistical analysis, and prepared figures and tables. SL provided guidance on statistical analysis. TD-S, SL, MJ, JS, and PP revised the manuscript. All authors contributed to the article and approved the submitted version.

## Conflict of Interest

SL is employed by deCODE genetics/Amgen, Inc. The remaining authors declare that the research was conducted in the absence of any commercial or financial relationships that could be construed as a potential conflict of interest. The reviewer ER-V declared a shared affiliation with the author TD-S to the handling editor at the time of review.

## Publisher’s Note

All claims expressed in this article are solely those of the authors and do not necessarily represent those of their affiliated organizations, or those of the publisher, the editors and the reviewers. Any product that may be evaluated in this article, or claim that may be made by its manufacturer, is not guaranteed or endorsed by the publisher.
